# Dental implants placement in paranoid squizofrenic patient with obsessive-compulsive disorder: A case report

**DOI:** 10.4317/jced.54356

**Published:** 2017-11-01

**Authors:** Lizett Castellanos-Cosano, José-Ramón Corcuera-Flores, María Mesa-Cabrera, José Cabrera-Domínguez, Daniel Torres-Lagares, Guillermo Machuca-Portillo

**Affiliations:** 1 Associate Professor, Department of Stomatology, School of Dentistry, University of Seville, Seville, Spain; 2 Master Special Care Dentistry, Department of Stomatology, School of Dentistry, University of Seville, Seville, Spain; 3 Professor and Chairman, Oral Surgery, Department of Stomatology, School of Dentistry, University of Seville, Seville, Spain; 4 Professor and Chairman, Special Care Dentistry, Department of Stomatology, School of Dentistry, University of Seville, Seville, Spain

## Abstract

**Background:**

Paranoid schizophrenia is a mental illness that involves no observable anatomical alteration. Main characteristic affects the personality of the individual, as well as areas of his own psychology.

**Case Report:**

A 33-year-old man with paranoid schizophrenia and obsessive-compulsive disorder in treatment with Haloperidol, Oxcarbazepine, Olanzapine and Seroquel is presented. Dental exploration showed widespread decay mostly cervical with numerous root fragments, agenesis of lateral incisors, impacted wisdom teeth, missing teeth and malocclusion. Treatment plan included restoration of teeth decay, extractions of root fragments and implant-supported prostheses in bilateral upper lateral incisors for aesthetics reason. A previous consultation with a psychiatric specialist was performed and no contraindication were observed. A preliminary radiological examination was performed previous dental treatment and implant placement. Due to patient refusal to replace dental abscenses with implants, inform consent was signed up from his parents. After local anesthesia, first implant was placed at upper right lateral positions (Straumann Bone Level Ø 3.3 mm, length 10 mm). Two weeks later a second implant was placed at upper left lateral position (Straumann Bone Level Ø 3.3 mm, length 12 mm). The patient showed no postoperative complications. After implant placement, the patient attended scheduled review appointments. The prosthesis was placed after a 3-month period of osseointegration.

**Conclusions:**

Implant placement can be considered a suitable option for people with mental disorders. A previous consultation with psychiatric specialists for conducting a good patient management is necessaire.

** Key words:**Paranoid schizophrenia, obsessive-compulsive disorder, dental implants.

## Introduction

Schizophrenia is considered to be a brain disorder characterized by diverse symptoms that might include hallucinations, delusions, lack of organized communication, a reduce capacity for planning, diminished motivation, and blunted affect (1). The incidence and prevalence of schizophrenia does not show a prominent location. Males are considered more risky to develop schizophrenia than females (2).

Schizophrenia and bipolar affective disorder are considered the most common disorders associated with severe mental illness. These conditions have proved to have a worse physical health with a life expectancy diminished when you compare to general population (3,4). First and second generations of antipsychotics drugs are frequently used in the treatment of these disorders. Both generation of drugs block brain dopamine receptors and can cause a negative effect on the patient’s ability to effectively brush their teeth and perform oral hygiene activities (5). These drugs have anticholinergic side effects as xerostomia, considered a major risk factor of dental caries. In addition, patients with dry mouth drink carbonated drinks more frequently, increasing the risk for caries (6).

Psychiatrists are not sufficiently alert to the risk and extent of dental pathology among their patients or to its psychological impact. In 1982, the World Health Organization published its recommendations for improving the socio-psychological aspects of oral health (7). Although oral health has improved throughout the global population, however psychiatric patients continue belonging to a minority group that have not reach this oral health state (8).

Dentists are generally unwilling to invest in complex conservative or rehabilitative treatments owing to the difficulty in treating patients with psychiatric disease (9). Advanced treatments such as dental implants placement in patients with mental disorders have not been described in the scientific literature. This is the first case report that describes a successful integrated oral treatment with implant placement in a patient with schizophrenia and obsessive-compulsive disorder.

## Case Report

A 33-year-old man with paranoid schizophrenia and obsessive-compulsive disorder was attended at the Faculty of Dentistry at the University of Seville. The patient was on treatment with Haloperidol, Oxcarbazepine, Olanzapine and Quetiapine (Seroquel®). Extraoral frontal exploration showed no proportional thirds, with a slight augmentation of the inferior facial third. The patient did not show teeth while was smiling due to a serious complex regard to his oral state. Sagital exploration showed a mandibular retrognatia.

**Figure 1:**
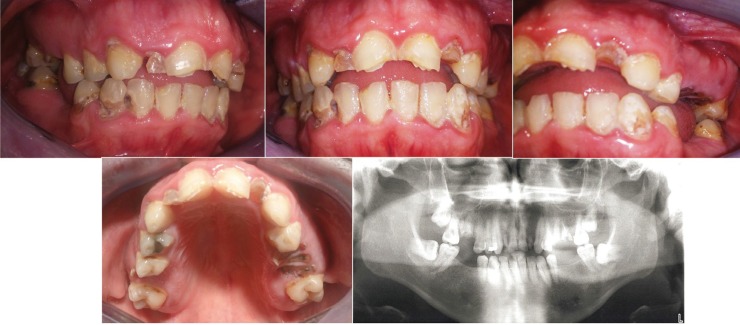
Initial oral state. (1) Right lateral intraoral photography, (2) Frontal intraoral photography, (3) Left lateral intraoral photography, (4) Upper occlusal intraoral photography and (5) Orthopantomography.

Dental exploration showed presence of temporary lateral incisors, widespread decay mostly cervical, numerous root fragments, impacted wisdom teeth, missing teeth and malocclusion. A preliminary radiological examination was performed and agenesis of permanent lateral incisors was observed (Fig. 1).

-Protocol Design

A previous consultation with his psychiatric specialist was performed and no contraindication were observed. Due to patient refusal to replace dental abscenses with implants, inform consent was signed up from his parents. Treatment plan included restoration of teeth decay, extractions of root fragments and implant-supported prostheses in bilateral upper lateral incisors for aesthetics reasons. A computarized axial tomography was realized previous implant placement with a radiological guide splint once a complete oral health state was achieve.

-Surgical Treatment

**Figure 2:**
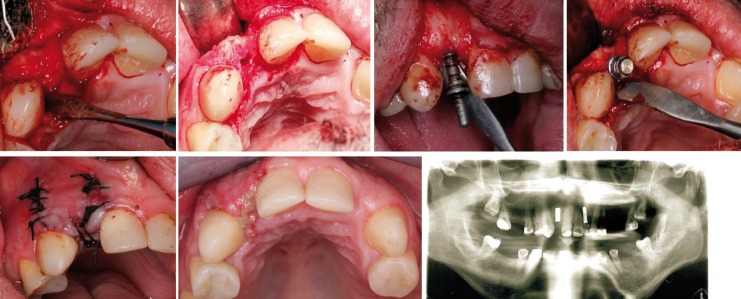
Surgical implant placement in upper right lateral incisor.

Surgical treatment was performed under local infiltrative anesthesia with vasoconstrictor (articaine plus 1:100,000 epinephrine) by periapical injection. A mucoperiosteal flap was made along the crestal bone of the edentulous space. First implant was placed at upper right lateral position (Straumann Bone Level Ø 3.3 mm, length 10 mm) (Fig. 2). Two weeks later a second implant was placed at upper left lateral position (Straumann Bone Level Ø 3.3 mm, length 12 mm) following the same protocol as upper right lateral implant placement. Implant closure screws were immersed in the flap in both implants surgeries to avoid self-injuries of the patient to a foreign body. Antibiotic and anti-inflammatory drugs were prescribed. The patient showed no postoperative complications. After implant placement, the patient attended scheduled review appointments.

-Prosthetic Treatment

After a 3-month period of osseointegration a second surgery was perform in order to expose both implant where healing abutments were placed. A week later implant impression were performed with an impression post screw-retained, with integral guide screw height of 10 mm in both implants.

**Figure 3:**
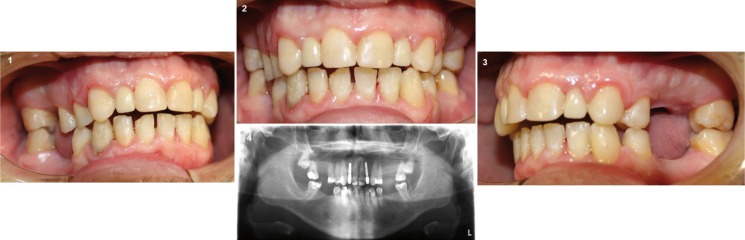
Follow up after 1 year. (1) Right lateral intraoral photography, (2) Frontal intraoral photography, (3) Left lateral intraoral photography and (4) Orthopantomography.

The prosthesis was placed and both patient and family were fully satisfied with the results. Follow up appointments were performed at 6 months and 1 year after coronal prosthesis implant placement (Fig. 3).

## Discussion

Our patient was under antipsychotics (Haloperidol, Olanzapine and Quetiapine) and antiepilectical (Oxcarbazepine) medications. The xerostomia consequently of antipsychotic drugs is often increased by the concomitant use of anticholinergic drugs, which are prescribed with high-potency antipsychotics in order to alleviate the Parkinsonian side effects of these psychiatric drugs (10). Clinical oral exploration showed numerous root fragments result of advance caries lesion without treatment. A widespread decay in mostly cervical of all teeth was also observed. This oral state may be a consequence of both concomitant poor oral hygiene and diminished saliva fluid. Once a complete oral health state was achieve, implant placement procedure were performs.

Dental implant placement in patients suffering from xerostomia have improved comfort and function of the patients (11,12). Although there are biochemical, immunological and microbiological changes in saliva composition that lead to an increase in the risk of infections (13). Antibiotic and anti-inflammatory drugs were prescribed after the surgical procedure in order to reduce this risk.

Our main concern about placing implants in a patient with paranoid schizophrenia and obsessive-compulsive disorder was due to the clinical characteristics of this psychiatric disorders. Numerous studies have proved that these patients lack the skills, physical dexterity and/or motivation to adopt and maintain good oral hygiene habits. Therefore, our long-term treatment would be compromised and could be taken over to failure. Moreover, it seems that these patients only visit the dentist when they have serious oral problems and do not bother with routine dental checks (14,15). Implant placement in our patient was not performed until oral hygiene educations was perform and successfully achieve in all visits appointments. Follow up after one year shows a good oral health and successful implant maintaining.

Today has not been eradicated the trend among dentists of continue performing dental extractions in psychiatric patients that suffer from dental pain, a new treatment goal in these patients should be to preserved and restored all the possible teeth (16). A clear preventive dental protocol should be established for psychiatric patients in all medical institutions (7). It has been shown that psychiatric patients who have restored their oral health have a better quality of life and improve their self-esteem. Furthermore, enhancing oral function contribute to an improvement in patients general health (17).

## Conclusions

This is the first article where is successfully described the placement of dental implants in a schizophrenia patient. A multidisciplinary approach should be perform in patients with mental disorders in order to fulfil both patients and professional objectives.
